# Optimizing In Vitro Propagation of *Haworthia truncata* Schönland Using Leaf, Root, and Inflorescence

**DOI:** 10.3390/plants14020212

**Published:** 2025-01-14

**Authors:** Leila Soleimani, Hassan Salehi, Taras Pasternak

**Affiliations:** 1 Department of Horticultural Science, School of Agriculture, Shiraz University, Shiraz 7144113131, Iran; soleimani9212@gmail.com; 2 Instituto de Bioingeniería, Universidad Miguel Hernández, 03202 Elche, Spain

**Keywords:** acclimatization, adventitious shoot regeneration, micropropagation, succulent plants, SEM, tissue culture

## Abstract

*Haworthia truncata*, a species native to South Africa, is characterized by its limited growth and scarcity, contributing to high production costs. Countries like China and Turkey are known for exporting *Haworthia* globally. Tissue culture offers an efficient method for mass-producing unique and beautiful species such as *H. truncata.* This study tested Murashige and Skoog (MS) basal media supplemented with various concentrations of IBA (0.05–1.5 mg/L), NAA (0.05–0.25 mg/L), and BA (0.25–1.5 mg/L) to promote shoot proliferation. MS medium without plant growth regulators (PGRs) was also tested as a control. Different explant types (leaf, root, and inflorescence) were analyzed for their potential in direct and indirect regeneration. Inflorescence explants showed the highest callus induction with 1.5 mg/L IBA, while optimal shoot proliferation occurred at 1 mg/L IBA. Callus induction was optimal for leaf explants with 0.05 mg/L NAA and 0.25 mg/L BA, and shoot proliferation was highest at 0.05 mg/L NAA and 1 mg/L BA. Root explants achieved maximum callus induction with 0.25 mg/L BA and 0.25 mg/L NAA, with the best shoot proliferation using 0.05 mg/L NAA and 1 mg/L BA. The highest rooting percentage of regenerated shoots was obtained on ½ MS medium with 1.5 mg/L IBA.

## 1. Introduction

*Haworthia* belongs to the Asphodelaceae family. It comprises 151 species that mainly exist in desert habitats of South African origin. As one of the ornamentals [1], it is a perennial, monocotyledonous plant and is kept in pots. Namibia, Southeast Africa, and Madagascar have major habitats where *Haworthia* species naturally grow. The plants are used for commercial cultivation and usually attract collectors [2,3]. Some species in this genus are invaluable because of their rarity, although they have a slow growth rate and propagate with difficulty. The price of the *Haworthia* genus on the retail market depends on its population, leaf form, leaf stripe, and leaf variegation [2]. Environments with plenty of shade are no problem for *Haworthia* species. As decorative plants, they have gained popularity across the globe and need minimum care for their growth and maintenance. Additionally, miniature gardens are finely decorated with the presence of *Haworthia* species. The transparent parts of the plant might appear as random spots, lines, and broad streaks. Based on this characteristic, they are characterized as window-leaved in vegetative terms [4,5]. *Haworthia* species are traditionally grown by seeds, leaf-cuttings, crown division, or propagules on inflorescences [2]. Due to its self-incompatibility, *Haworthia* is often propagated by leaf cuttings. However, since mother plants of *Haworthia* species generate few offshoots, the leaf-cutting method for propagation is rather time-consuming [6,7]. Therefore, micropropagation techniques are reportedly apt for the optimal propagation of *Haworthia* species. The approach permits plant multiplication without seasonal and climatic limits and occurs broadly; it is deemed essential for breeding decorative and landscape plants [4,8]. *H. truncata* is a small leaf succulent characterized by oppositely oriented, truncated leaves. This plant grows flat on the ground with fleshy leaves only emerging above the soil surface. The little, white, aloe-like blooms are produced mostly in late spring or early fall on long, thin stalks 15 cm long [9].

Due to obstacles in seed production, *Haworthia* species have benefited significantly from micropropagation. This method enables the regeneration of plants from inflorescence structures [10], leaves [7], and ovarian segments [10,11]. Several factors influence micropropagation efficiency, including, but not limited to, nutrient availability, explant type, plant growth regulators, media supplements, light intensity, and duration. In certain cases, callus induction has been successfully achieved from different plant parts [10,12]. In vitro, plantlets have also been regenerated from all organs of this genus [4,6,7,13,14,15,16]. In vitro propagation of *Haworthia* is important since these species serve as a source of important secondary metabolites [17].

Previous research on *Haworthia* species successfully produced plantlets from inflorescence explants [10,11,13], leaves [15], and ovary walls [18]. Nonetheless, these research attempts did not employ root explants for propagating these plants in vitro, which may be because of the belief in high infection among root explants of most plants. Micropropagating this unique and expensive plant with roots or other parts would be a great success. So far, no research has considered the in vitro propagation of *H*. *truncata* from leaf, root, or inflorescence explants simultaneously. Accordingly, this research designed a quick, effective micro-propagation protocol to propagate *H. truncata* with the aim of mass production and provide a new method to overcome obstacles to the reproduction of *H. truncata*. The procedure employs various explants from this valuable ornamental plant.

## 2. Results

### 2.1. Callus Induction and Shoot Induction from Inflorescence Explants

**Figure 1:**
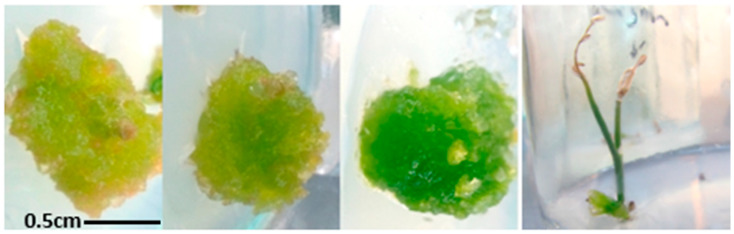
Quality of calluses obtained from inflorescences. From right to left are the best quality (grade 1) and the qualities of grades 2 and 3, respectively. The last one is the initial stage of the callus obtained.

Few inflorescence explants survived due to the high sensitivity of the tissue to sterilization. The highest survival rate was observed in the explants taken from the flower and the axis of the inflorescence at the terminal part, with floral buds performing optimally in callus induction (Figure 1). The average diameter of the induced calluses from the flower explants was 1.11 cm. The largest callus diameter occurred in response to 1 and 1.5 mg/L of IBA. However, the best callus quality was achieved with 0.5 mg/L of IBA and 1 mg/L of NAA, with fresh green color, and hard and crispy texture, showing a significant difference between treatments (*p* ≤ 0.05) in quality (color (Color: 0 (White), 1 (Yellow), 2 (Yellow-green), 3 (Light green), 4 (Dark green); and hardness (Hardness: 0 (Very soft, Water-soaked), 1 (Soft), 2 (Semi-hard), 3 (Hard), 4 (Very hard)). The lowest quality callus was produced under 1 mg/L of BA. Overall, calluses from inflorescence explants cultured on a medium containing IBA had better quality and growth compared to those cultured on a medium containing BA (the hardness was higher, the color was closer to fresh green, and the diameter was greater) (Table 1).

NAA and 2,4-D were also used in different experiments which results were not good enough to represent. A concentration of 1 mg/L of NAA was sufficient to produce the desirable color and firmness of the callus. While 0.5 and 1 mg/L of 2,4-D were used for callus formation, the resulting calluses were not suitable in terms of quality and quantity. Calluses grown on IBA demonstrated the best quality and regeneration ability, followed by those treated with NAA and 2,4-D. Callus mass which was fresh green in color and kind of fresh, crispy, and hard texture, potentially had regenerative potential.

After the callus formation from the flower explant, the obtained calli were subcultured in BA- and IBA-enriched media to investigate the number and length of produced shoots. Callus samples were divided into 0.3 cm pieces for subculturing which concluded that IBA (1 mg/L) was the best treatment (Table 2).

**Figure 2:**
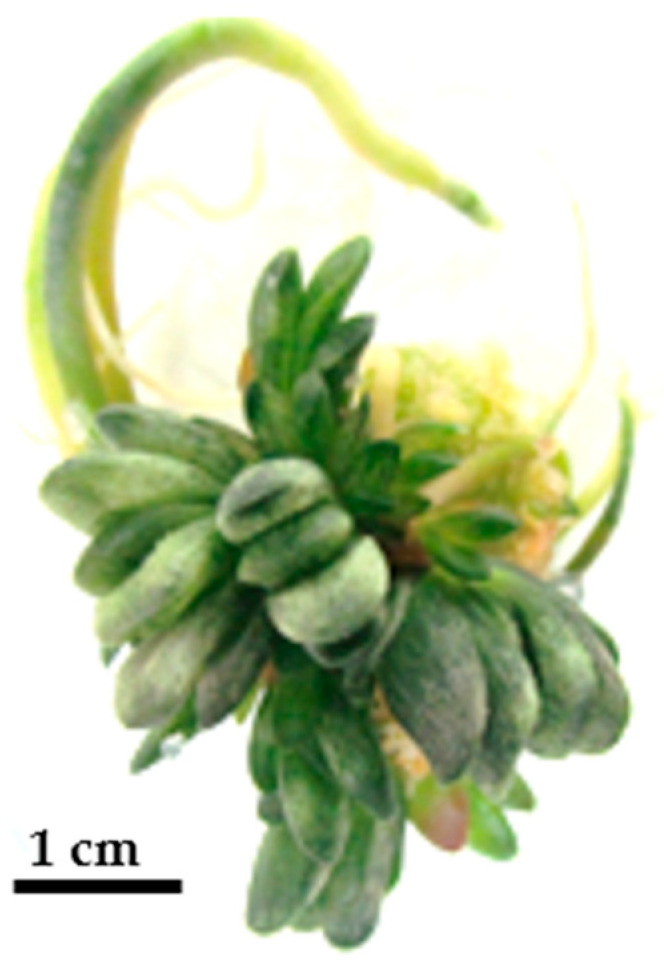
Bicolor plantlets on BA-enriched media (1 mg/L).

Bicolor plantlets were produced on BA-enriched media (1 mg/L) (Figure 2).

**Figure 3:**
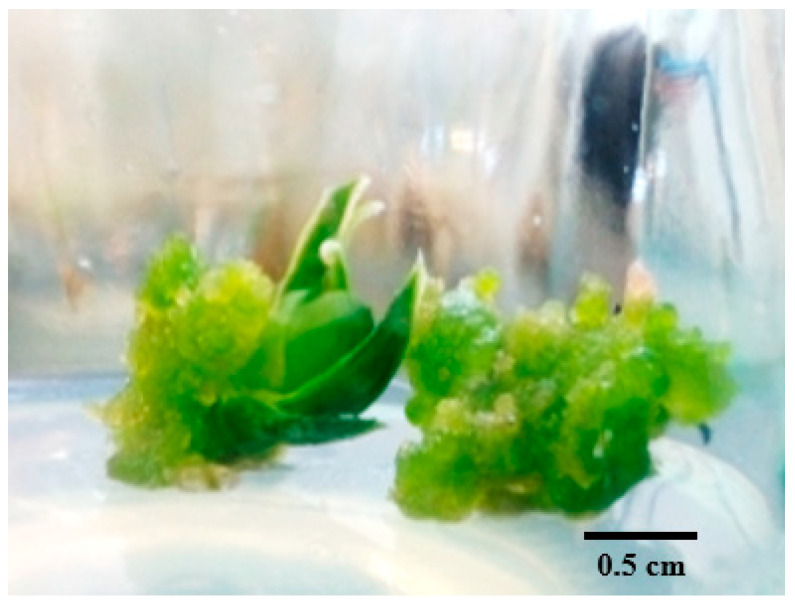
Callus-mediated shoot-regeneration on IBA-enriched MS media (1 mg/L).

**Figure 4:**
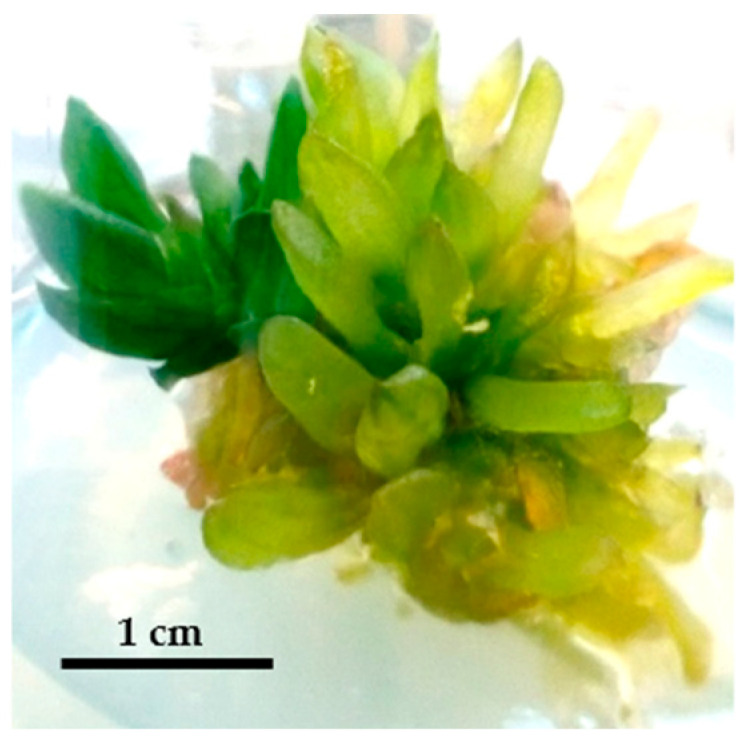
Vitrification on the medium containing BA, 1 mg/L.

On average, there were 5 plantlets) in each subculture. The average size of the plantlets was 0.4 cm. More plantlets were obtained from IBA-enriched (1 mg/L) media. The largest size of plantlets (*p* ≤ 0.01) also appeared in the same treatment (Figure 3), with a low percentage of vitrification (*p* ≤ 0.01). The highest deformity with high vitrification was observed with BA 1 mg/L (Figure 4). The lowest amount of plantlet deformity occurred in response to IBA (0 to 1.5 mg/L), which differed significantly from the others (*p* ≤ 0.01) (Table 3). Vitrification in the plantlets disappeared after rooting, during adaptation.

### 2.2. Callus Induction and Shoot Proliferation in Leaf Explants

Data collection was performed 8 weeks after the start of the experiment, with most treatments showing noticeable effects between five and seven weeks. Explants that did not respond within this period generally showed weak growth. Although the number of plantlets producing calli in the control group was similar to those treated with Plant Growth Regulators (PGRs), the callus formation in the PGR treatments was lower than in the control group. In contrast, when treated with BA (1.5 mg/L) and IBA (0.05 mg/L), callus formation was limited, with the average callus formation rate in leaf explants being 38.49%.

Shoot proliferation was also observed in the control group, but several treatments showed lower shoot proliferation rates compared to the control. Notably, no shoot proliferation occurred in treatments with BA (0.25 mg/L), IBA (0.25 mg/L), or BA (1.5 mg/L) combined with NAA (0.05 mg/L). However, when using a combination of BA (0.25 mg/L) and NAA (0.25 mg/L), shoot proliferation produced significantly to 58.33%, which was a substantial improvement compared to the control.

**Figure 5:**
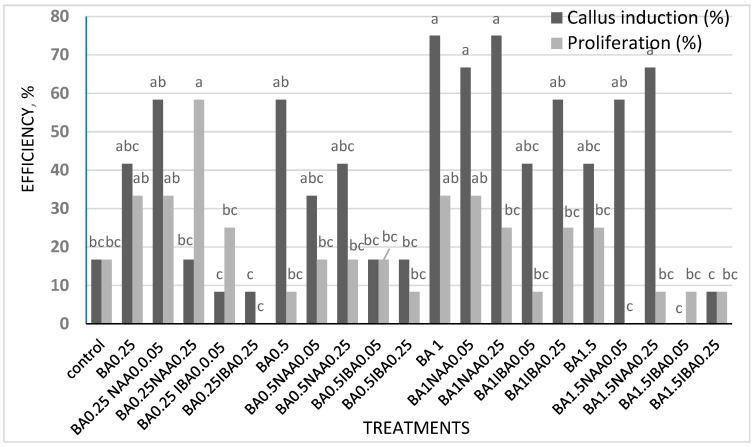
Shoot proliferation and callus induction percentage of leaf explants. Similar letters denote insignificant differences per column (LSD test, *p* < 0.05).

On average, the shoot proliferation rate in leaf explants was 19.44% (as shown in Figure 5).

### 2.3. Callus Formation and Shoot Proliferation Analysis

**Figure 6:**
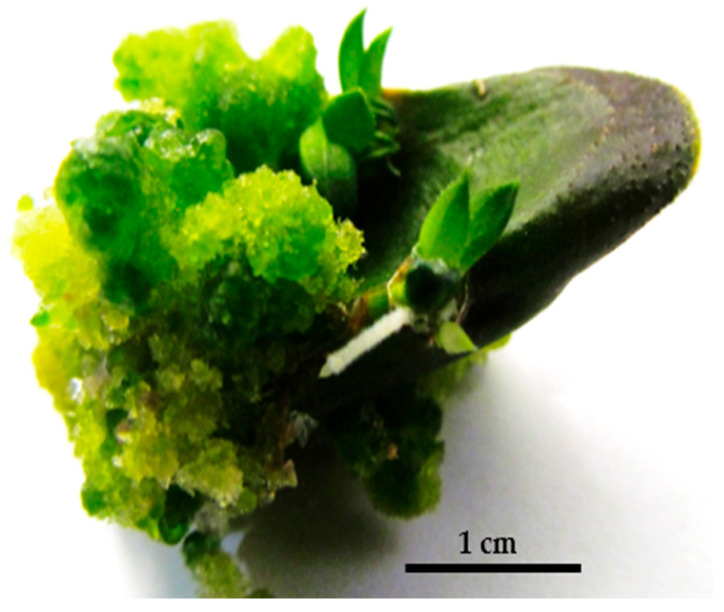
The largest callus diameter was observed with 0.25 mg/L BA and 0.05 mg/L NAA.

Callus formation and shoot proliferation characteristics were assessed in the explants that responded to the treatments. As shown in Table 4, the largest callus diameter was observed with 0.25 mg/L BA and 0.05 mg/L NAA, resulting in 58.33% callus formation (Figure 6) and an average diameter of 1.84 cm. The use of 1 mg/L BA led to a higher callus formation rate of 75%, with an average diameter of 1.74 cm. However, the quality of callus at 0.25 mg/L BA with 0.05 mg/L NAA was superior in terms of texture and overall quality. For callus color and firmness, the best results were achieved with 1.5 mg/L BA, producing calli with an average diameter of 0.89 cm (*p* ≤ 0.01). Overall, BA (1 mg/L), whether combined with auxins or used alone, resulted in a satisfactory callus formation rate. When using similar concentrations of BA and NAA, the conditions were more favorable for callus formation.

**Figure 7:**
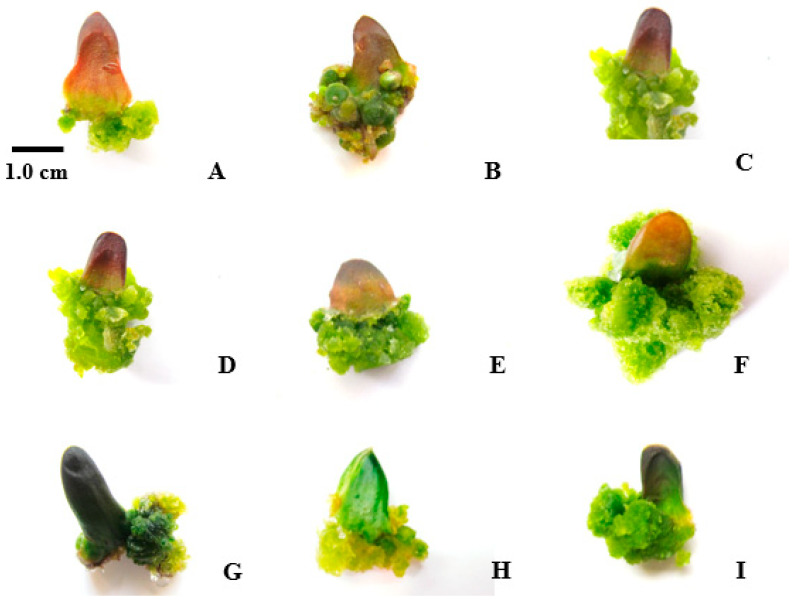
Callus induction from leaf explants with PGRs: control (**A**), BA 1.5 mg/L with NAA 0.25 (**B**), BA 1.5 with NAA 0.05 (**C**), BA 1 (**D**), BA 0.5 with IBA 0.05 (**E**), BA 0.25 with NAA 0.05 (**F**), BA 0.25 with IBA 0.05 (**G**), BA 0.25 with IBA 0.25 (**H**), BA 0.25 (**I**).

In terms of callus diameter, BA without auxin produced better results as the concentration increased. Regarding callus color and firmness, the best outcomes were observed when BA was used without auxin, with 1.5 mg/L BA yielding optimal results (Figure 7).

### 2.4. Shoot Proliferation

**Figure 8:**
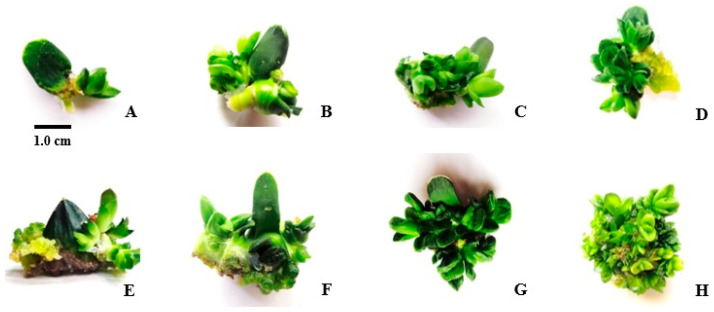
Leaf regeneration from leaf explants using various PGR concentrations: BA (0.5) (**A**), control (**B**), BA (1) (**C**), BA (0.5) with NAA (0.25) (**D**), BA (0.25) with IBA (0.05) (**E**), BA (0.25) (**F**), BA (1) with NAA (0.05) (**G**), BA (1.5) with IBA (0.05) (**H**).

In the first experiment, leaf tissue was cultivated both horizontally and vertically. However, the horizontal explants did not survive. The vertically grown explants, with a minimum size of 0.5 cm, were suitable for further growth. The combination of BA (0.25 mg/L) and NAA (0.25 mg/L) resulted in the highest shoot proliferation rate of 58.33%. Using BA (1.5 mg/L) with IBA (0.05 mg/L) led to an 8.33% shoot proliferation rate, with an average of about 15 plantlets. In comparison, BA (1 mg/L) mixed with NAA (0.05 mg/L) produced a 33.33% shoot proliferation rate. The combination of BA (0.5 mg/L) with IBA (0.05 mg/L) caused 16.67% proliferation, with averages of 10.33 and 8.33 plantlets, respectively. The plantlets from these treatments were larger and had more leaves, indicating their effectiveness (*p* ≤ 0.01). Overall, treatments with BA alone or BA mixed with NAA produced better results (shoot count and shoot length) compared to BA mixed with IBA and BA (1.5 mg/L) without auxin. Furthermore, using exogenous auxins for shoot proliferation is not recommended, as the results showed low shoot proliferation rates. Even when a significant number of plantlets was produced, their size was below average. In terms of plantlet count and leaf number, increasing auxin concentrations from 0.25 mg/L to 1 mg/L resulted in a higher number of plantlets. However, in treatments without auxin or those mixed with auxin, the average size and leaf count of the plantlets decreased. Increasing BA from 0.25 mg/L to 1 mg/L, either mixed with NAA or used alone, led to better results in plantlet count compared to combinations with IBA (Figure 8).

**Figure 9:**
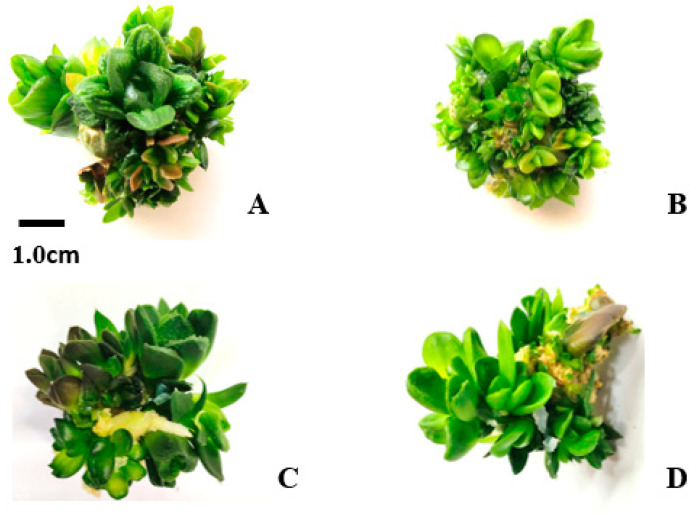
Shoot regeneration from leaf explants using various PGR concentrations after 3 months: BA (1) with IBA (0.05) (**A**), BA (0.5) with IBA (0.05) (**B**), BA (1.5) with NAA (0.25) (**C**), BA (1.5) (**D**).

Interestingly, an increase in plantlet count was associated with smaller plantlets. When cytokinin (BA) was combined with exogenous auxins, plantlets were generally larger compared to the case when BA was used without auxins (Table 5). In some treatments, such as BA (1 mg/L), irregular leaf arrangements and deformities were observed because of endogenous auxin induction (Figure 9).

### 2.5. Callus Induction and Direct Shoot Proliferation from Root Explants

**Figure 10:**
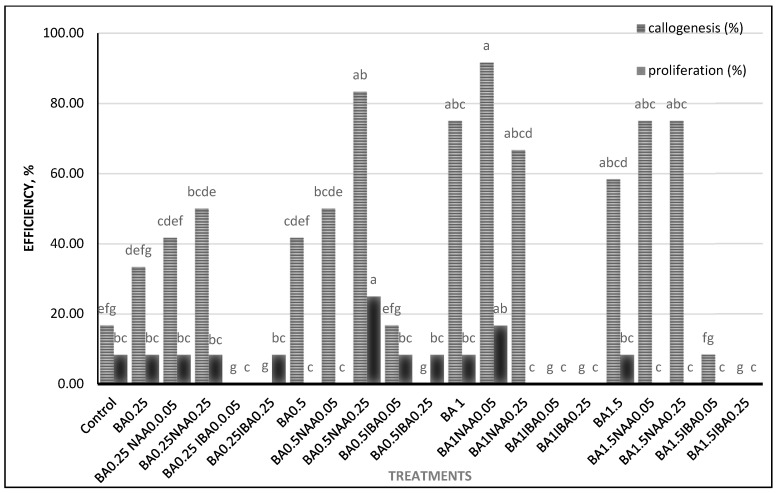
Shoot proliferation and callus induction percentage of root explants. Similar letters denote insignificant differences per column (LSD test, *p* < 0.05).

Since sucker growth is one of the ways to propagate this plant, it is expected that the root explant can be used for micropropagation. The results showed that in the root explant, shoot proliferation takes place with a low percentage compared to the leaf explant, and the explants with at least 1 cm had a better response. Root explants were cultured in 20 treatments similar to leaf explants along with the control treatment, which had callus induction in most of the treatments, but few treatments showed shoot proliferation (Figure 10).

**Figure 11:**
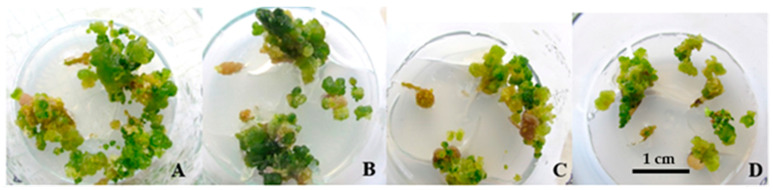
Callus induction from root explants using PGRs: BA (1) with NAA (0.05) (**A**), BA (0.5) with NAA (0.05) (**B**), BA (1) (**C**), BA (1.5) with NAA (0.05) (**D**).

The results indicated that the control treatment (MS medium without any PGRs induction) produced calli with larger diameters compared to the treatment groups, but the quality of these calli was notably lower. The highest percentage of callus induction (91.67%) was observed with BA (1 mg/L) combined with NAA (0.05 mg/L), though the quality of the callus remained low. Treatments with BA (0.25 mg/L) combined with NAA (0.25 mg/L), as well as BA (0.5 mg/L) with NAA (0.05 mg/L), resulted in 50% callus induction. These combinations showed significantly better performance in terms of both callus diameter and quality compared to other treatments (*p* ≤ 0.01) (Table 6). Overall, combinations of BA and NAA resulted in higher percentages of callus induction, larger callus diameters, and better firmness compared to BA and IBA combinations. Additionally, BA without auxin also led to better outcomes in callus formation, diameter, and firmness than the BA and IBA combinations. The color index for the BA and NAA combinations was superior to that of the other treatments (Figure 11).

**Figure 12:**
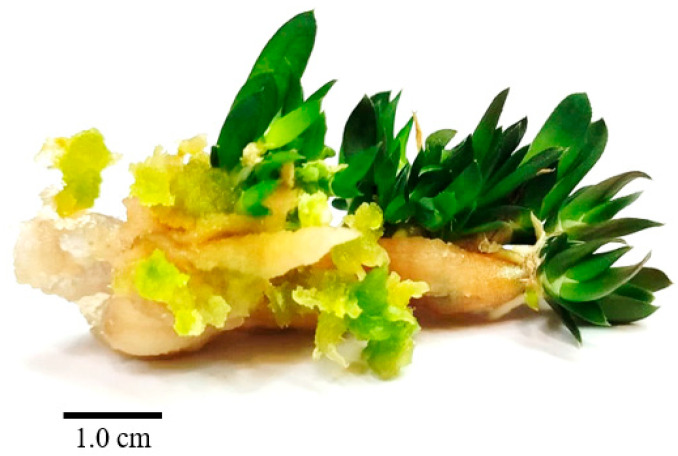
Shoot regeneration from root explants using BA (1) and NAA (0.05).

**Figure 13:**
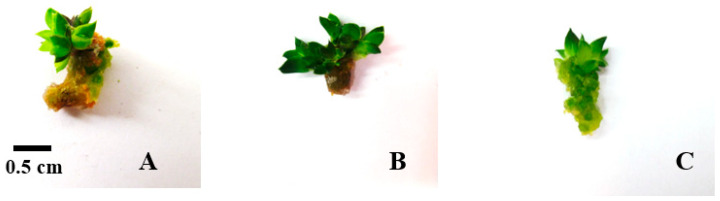
Shoot regeneration from root explants using PGRs: BA (0.5) with NAA (0.25) (**A**), BA (0.5) with IBA (0.05) (**B**), BA (0.5) with NAA (0.25) (**C**).

The findings also showed that longer and thicker explants performed better. Optimal results were achieved when roots were 4 cm in length or used in their entirety (without cutting), retaining their normal size, and avoiding the removal of the meristem or proximal areas. In some cases, multiple plantlets were generated when the roots grew long and vigorous (Figure 12). Roots from younger tissues resulted in more successful callus formation (Figure 13), while roots with firmer tissues led to higher levels of shoot proliferation.

**Figure 14:**
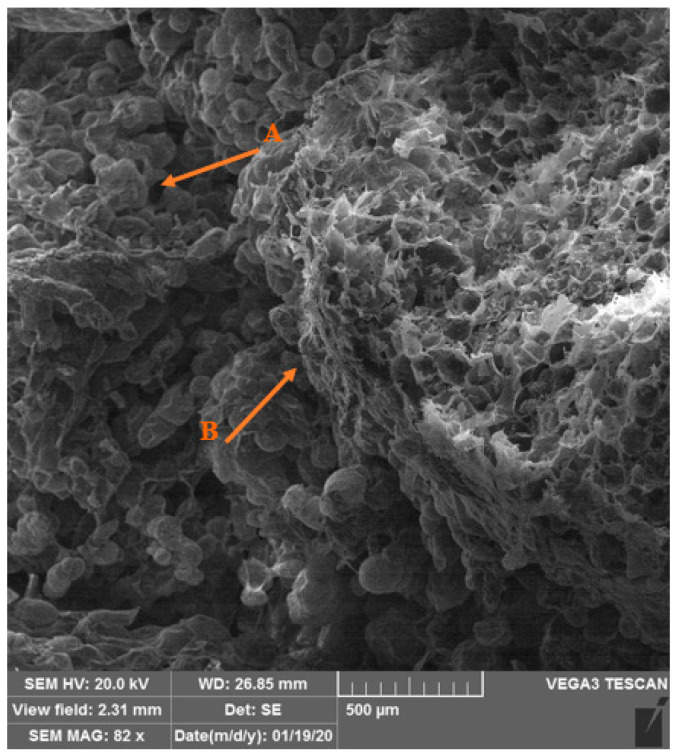
Scanning electron micrographs of the callus induction from the root, callus texture (A), and root texture (B) at 82 magnifications.

**Figure 15:**
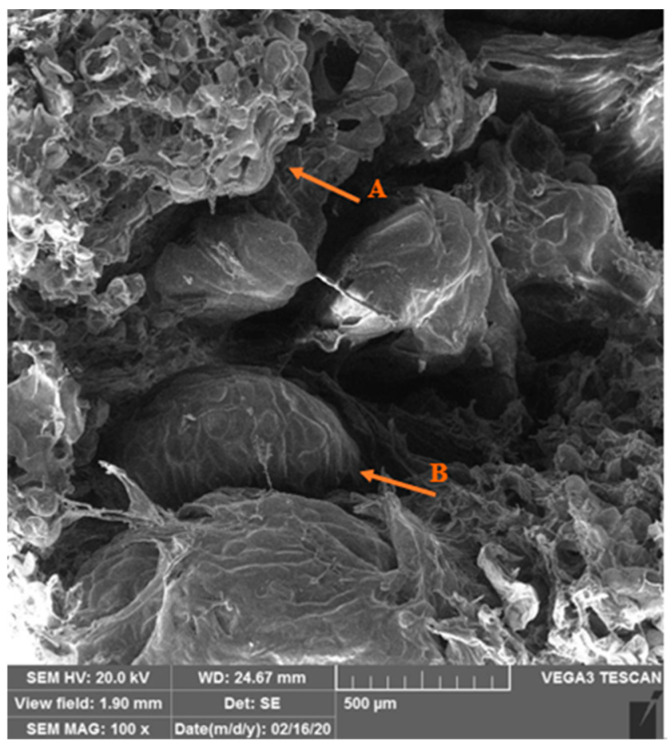
Scanning electron micrographs of the shoot regeneration from the root, root texture (A), and new shoot texture (B) at 100 magnifications.

Scanning electron micrographs of the callus induction from the root, callus texture (A), and root texture (B) are shown in Figure 14, and the micrographs of the shoot regeneration from the root, root texture (A), and new shoot texture (B) is shown in Figure 15.

The average plantlet count was 3.6, the average shoot length was 0.39 cm, and the leaf count averaged 2.62 (Table 7). Accordingly, 0.5 mg/L BA and 0.25 mg/L NAA had the highest percentage of shoot proliferation (25%). BA (1 mg/L) mixed with NAA (0.05 mg/L) led to 16.66% shoot regeneration and the largest plantlet count (7.5 plantlets per explant on average). The control treatment had the highest plantlet size (1 cm on average). BA (0.25 mg/L) mixed with IBA (0.25 mg/L) produced the largest plantlets (0.37 cm on average). All parameters had significant differences (*p* ≤ 0.01). The leaf count was maximum in the control because fewer plantlets meant larger plantlets with more leaves. In general, the mixed treatments of NAA and BA, compared to BA without auxin, showed better results in the plantlet count index, even though BA (0.25 mg/L) mixed with IBA (0.25 mg/L) had better results than BA (0.25 mg/L). The average plantlet size in BA treatments (0.5 mg/L) resulted in more acceptable plantlets than the BA treatment (0.25 mg/L). However, the plantlet size decreased in response to BA treatments (1 and 1.5 mg/L).

### 2.6. Rooting

**Figure 16:**
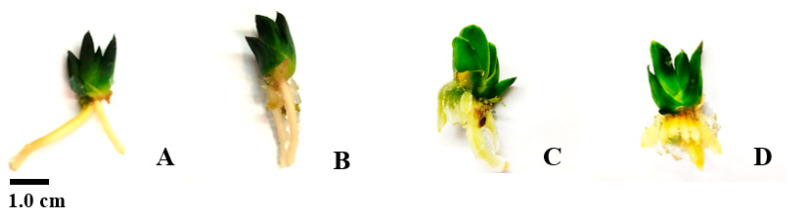
Rooting as affected by NAA (0.2 mg/L) (**A**), NAA (1 mg/L) (**B**), IBA (0.2 mg/L) (**C**), and IBA (1 mg/L) (**D**).

**Figure 17:**
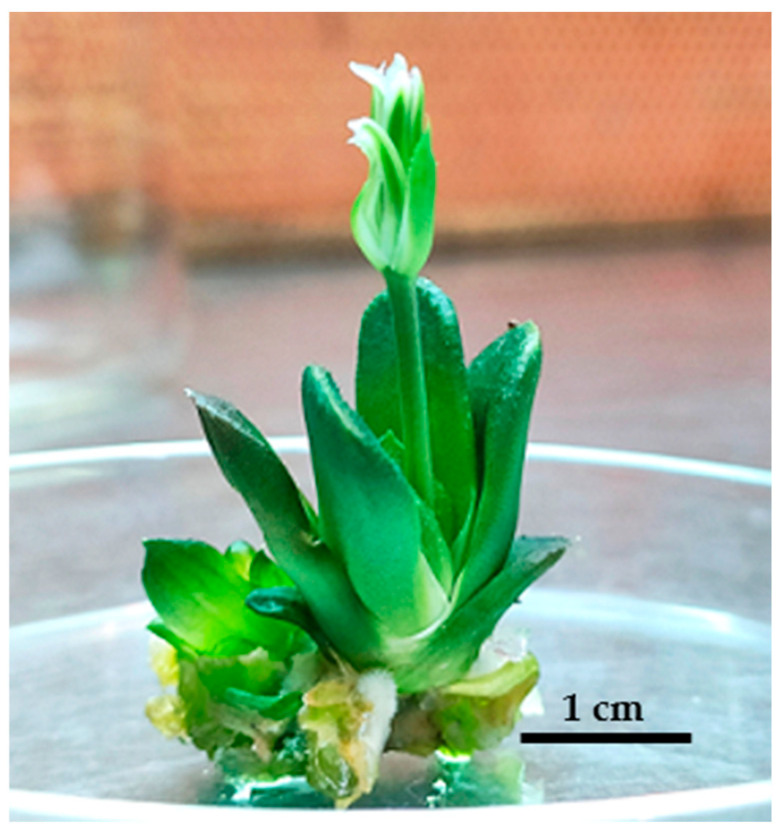
Flowering as affected by IBA (0.1 mg/L).

About 1 cm plantlets were used for rooting in a semi-concentrated MS medium and data collection was done after eight weeks. The results showed that the average root count differed significantly depending on the PGR treatment (*p* ≤ 0.05), especially regarding 0.1 and 0.2 mg/L IBA, compared to NAA at the same concentrations (Table 8). IBA (1 mg/L) led to greater root shoot proliferation than in the other treatments (Figure 16), although without becoming significantly different. The root length was significantly less (*p* ≤ 0.05) in the absence of PGRs (Table 8). The root diameter index did not differ significantly in any case (*p* < 0.05).). IBA (1.5 mg/L) led to maximum rooting (100%) and outcompeted all other treatments (*p* ≤ 0.01) (Similar letters denote insignificant differences per column (LSD test, *p* < 0.05) Table 9).In one of the plants treated with IBA (0.1 mg/L), flowering was seen almost a month after the treatment (Figure 17).

### 2.7. Acclimatization

**Figure 18:**
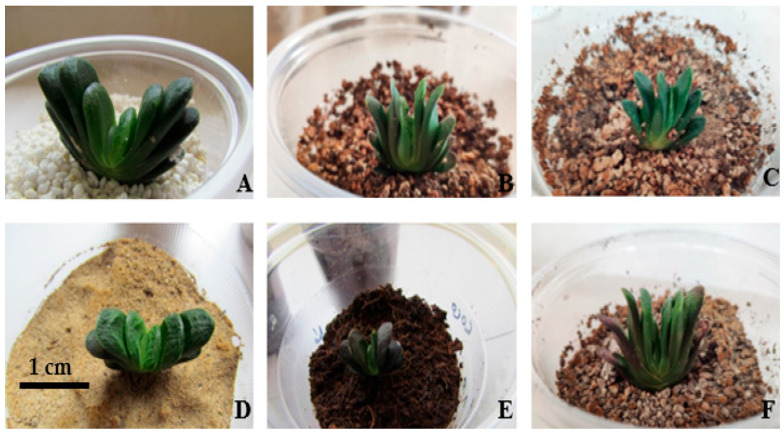
Acclimatization of plantlets in different substrates: perlite (**A**), peat moss + perlite (**B**), sand + peat moss + perlite (**C**), sand (**D**), cocopeat (**E**), peat moss + perlite + vermiculite (**F**).

After the rooting stage, well-rooted plantlets were cultivated in cocopeat, perlite, sand, peat moss, and vermiculite in single and mixed form (in equal proportions) (Figure 18). Eight weeks after transfer and adaptation, data collection was done (before the adaptation of these parameters was measured and after 10 weeks it was measured again and the difference between the two was used as data). The highest increase in root size with an average of 2.4 cm occurred in cocopeat and perlite with an equal ratio, which had a significant difference from other combinations (*p* ≤ 0.01). The root count increased substantially and reached 3.75 per plantlet. The leaf count reached a maximum of 3 leaves per plantlet on average in the perlite medium, which had a significant difference (*p* ≤ 0.01). Regarding other traits, such as plant size, secondary roots, and decay, no significant difference was observed (Table 10).

The cocopeat-perlite medium led to 40.47% rootlet production, which was the highest rate among all treatments, and the lowest percentage of root rot was 0% in the sand medium (Table 10). The percentage of survival in all adaptation treatments was 100%. After two months, peat moss and sand (3:1) were used in the greenhouse. After three months, the plants became marketable.

## 3. Discussion

*H. truncata* is an ornamental plant with nutritional and medicinal properties. Due to its slow growth and low natural reproduction rate, micropropagation offers an effective method for mass production under tissue culture conditions.

Our tissue culture experiments with *H. truncata* demonstrated that this plant is highly suitable for in vitro micropropagation. All plant organs can serve as explants. The best callus formation was observed in inflorescence explants treated with IBA (1.5 mg/L). While IBA (0.5–1 mg/L) produced high-quality calluses and BA (0.5 mg/L) was comparable in terms of size and quality, most of the results were favorable. However, treatments with NAA and 2,4-D produced weaker calli, less ready for organogenesis. This is likely due to NAA and 2,4-D being synthetic auxins, while IBA and IAA are natural auxins, which are generally more effective.

One notable finding was the formation of callus in cytokinin treatment without auxin, which is rare. Callus shoot proliferation was optimal with IBA (1 mg/L), in line with previous studies using IAA (0.1 mg/L) without cytokinin. Although shoot proliferation rates were good, a significant percentage of new shoot deformation was observed. Overall, calluses formed in IBA had better regenerative abilities compared to those produced in NAA or 2,4-D.

In a related experiment, NAA (0.1 mg/L) combined with BA (0.5 mg/L) was the most effective for callus induction in *Haworthia cooperi*, while another study showed that BA (1 mg/L) combined with NAA (0.4 mg/L) resulted in optimal shoot development [19]. This suggests that exogenous auxins without endogenous auxin (induced by exogenous cytokinins) can still promote shoot proliferation in some monocot species, highlighting the versatility of exogenous plant growth regulators.

High concentrations of exogenous BA induced high endogenous auxin production which led to rapid vacuolar expansion and vitrification. Moreover, callus formation (unorganized growth) formed in vitro may lead to somaclonal variation. In our study, 0.25 mg/L BA combined with 0.05 mg/L NAA produced 58.33% callus formation, corroborating previous findings. While cytokinins are generally less effective than auxins in callus formation, in this case, a higher ratio of BA to NAA produced better results. Interestingly, BA treatments without auxin also yielded satisfactory outcomes, demonstrating that cytokinins can effectively stimulate cellular shoot proliferation and promote proper callus growth in *H. truncata*.

The best shoot induction from the leaves was 1 mg/L BA in combination with 0.05 mg/L NAA with 33.33% shoot proliferation, confirming earlier research on *H. cooperi* [20], *H. turgida* [4], and *H. margaritifera* [21] by shoot proliferation treatments from leaf explants, with higher ratios of endogenous auxin-induced by cytokinin, resulting in better shoot regeneration. Also, another research was done on *H. truncata* using leaf segments [22]. The best treatments in plantlet size were the combination of BA with auxin (compared to BA without auxin), which indicates that plantlet growth increased by auxins. Another noteworthy point was the better result of the mixture of BA and NAA on the plantlet count index than the mixture of BA and IBA. NAA is a synthetic auxin that is stronger than IBA and can be a reason for its better effect.

The best callus induction from the root was created in 0.5 mg/L BA mixed with 0.05 mg/L NAA with 50% callus induction. Mixtures of BA and NAA led to optimum results. Similar to the leaf tissue with higher BA concentration than auxin, better callus induction indicated the positive effect of cytokinin on indirect regeneration. There is no report about callus formation of root explant before this research. The maximum percentage of callus induction was higher in the root explants than in the leaf. Based on the observations, the highest growth rate was related to the callus obtained from the inflorescence compared to the callus obtained from the leaves and roots in most treatments, callus formation started in leaf explants about four to five weeks after cultivation, and in roots, it took about five weeks, but in inflorescences, it took two to three weeks for callus induction to start. In this research, SEM documents to observe and confirm the results of experiments were saved.

The low growth rate of this plant is more noticeable in its leaves and roots than in its flowers. This difference can be attributed to the accumulation of auxin in flowers and inflorescences. Auxin movement in the roots is slower than in the shoots. Since auxin is a key factor in the stimulation and growth of the callus, the inflorescence has a higher speed of stimulation and growth of the callus than other organs. Shoot proliferation and callus induction were observed in leaf and root explants in the control treatment. Sometimes, the high internal level of biochemical compounds, especially auxin, causes such reactions. Since plant growth regulators are exogenous, their effects may vary depending on external stimuli, leading to a decrease or stop of natural growth. Due to possible errors, the location of the explant, and environmental conditions, contradictory differences can be observed.

The *Haworthia genus* has been rooted in different reports in full and semi-concentrated MS media, even where PGAs did not function optimally. In this experiment, 1.5 mg/L IBA caused optimal rooting. In IBA dosages, the number of roots increased compared to NAA. However, in NAA treatments, increasing the concentration reduced the rooting percentage. Where the MS medium was semi-concentrated in the control, rooting was also seen. Earlier rooting was observed in NAA treatments than in IBA. Ten days after treatment, rooting occurred with 0.2 mg/L NAA and IBA after 14 days. The first rooting occurred with 0.1 mg/L IBA. In line with earlier research [4,7] NAA (0.1 mg/L) initiated rooting optimally in five species of this genus. NAA (0.05 mg/L) initiated optimal rooting in *H. turgida*. Half-concentrated MS media in *H. cooperi* appeared effective with 2 mg/L NAA and 0.5 mg/L IBA [22]. A similar condition with NAA and IBA (0.1 mg/L) resulted in optimal rooting in *H. margaritifera* [2]. For rooting, 0.1 mg/L IBA can yield efficient results. Moreover, investigating the micropropagation of *Haworthia limifolia* showed the best rooting on IBA (1.5 mg/L) [23]. In the control treatment, compared to the auxin treatments, the root length was longer, because the presence of auxin is useful in stimulating rooting, but prevented shoot growth and elongation. In general, *H. truncata* is an herbaceous plant with fleshy leaves and relatively easy rooting in vitro. The plant was estimated to be rooted in the control environment as well, which can be related to its herbaceous nature. Flowering in an auxin-containing environment was an interesting event, which could be because auxin is effective in the initiation of flowering and the growth of the flowering stem. In normal in vivo conditions, flowering depends on temperature (18–23 °C) and is usually observed in early spring and early autumn. In vitro flowering in this plant is attributable to subculture stress, suitable temperature, and auxin effects at the same time.

In this research, perlite was the best medium for plantlet acclimation. It caused the greatest increase in leaf count and rooting, with a low incidence of root rot (13.54%). The sand medium increased root size, and the percentage of root rot was zero, leading to a 100% survival rate. Others used peat moss mixed with perlite for five different species of *Haworthia* [7]. A mixture of soil and vermicelli was also used in *H. turgida* and the survival rate was 91.6% [4]. In the first stages, after being transferred to the greenhouse, the plant showed a little weakness, but with the prevention of strong sunlight and Bioradicant fertilizer (0.5 mL per pot), rooting and establishment in the environment of the greenhouse were improved. According to the observations, it is recommended to use washed perlite or river sand to acclimatize the tissue culture plants and for one month, MS medium with one-third concentration alternately with distilled water for irrigation should be done, because this plant is sensitive to root rot and rots quickly in an unsuitable substrate.

## 4. Materials and Methods

Pot plants of *H. truncata* ‘Lime Green’ with an age of at least one year old were purchased from a commercial greenhouse at Veterinary School, Shiraz University, Shiraz, Iran. The plants were relocated to a greenhouse at the College of Agriculture, Shiraz University, in March 2019.

**Figure 19:**
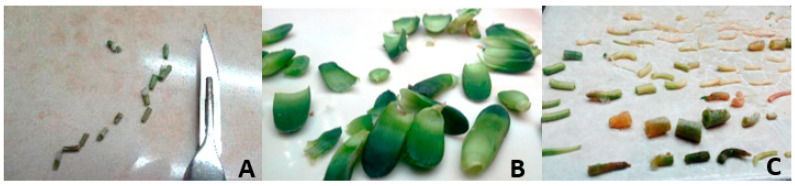
Explants just before culturing on medium: inflorescence (**A**), leaf (**B**), root (**C**).

Disinfection of root and leaf: Fresh leaves and roots were used as explants. Root and leaf explants both were disinfected with benomyl fungicide (5 g/L) for 30 min. After washing the leaves with tap water, a household liquid detergent (Pril) was added and maintained in the solution for the next 20 min. Then, 70% ethanol was used to disinfect the leaf samples for one minute and then they were washed repeatedly with distilled water. Domestos 30% (chlorox 5%) was used in combination with 0.05% Tween 20 for 20 min to sterilize the explants. Thereafter the samples were washed with sterile water and drained in sterile conditions. Subsequent experiments required that the leaf samples be dissected into 0.5–1.0 cm pieces using a sterile sharp blade (Figure 19). Ethanol 70% for 1 min and 30% Domestos detergent for 15 min were used to disinfect root explants. The roots were disinfected using 70% ethanol.

Disinfection of the inflorescence: The inflorescence was sterilized with 3% of chlorox. Contamination was controlled with 70% ethanol for 5 s and 5% chlorox for 9 min.

Environmental condition: The culture medium was mainly MS, comprising vitamins, baseline salts, 0.8% plant-agar, and sucrose (3%). PGRs entered the culture media before pH adjustment. The medium pH was adjusted to 5.8 and the jars were autoclaved (121 °C) for 60 min. All cultured samples were grown under 16-h photoperiod (24 ± 1 °C), and 30 μmol·m^–2^·s^–1^ light intensity. Each experiment consisted of 16 explants and had four replicates.

Subculturing on MS supplemented by PGRs: Inflorescence segments were sampled to induce callus on MS culture media enriched with IBA (0.5, 1, and 1.5 mg/L), together with the same concentrations of BA. After obtaining the callus, it was segmented into 0.3–0.4 cm^2^ pieces and subcultured onto an enriched MS medium, comprising IBA and BA at the same three concentrations (mentioned earlier) for adventitious-shoot induction and callus induction. Root and leaf explants were cultured on the same treatments with a variety of concentrations of IBA and NAA with BA as mentioned in Figure 5 and Figure 10. The callus induction percentage and callus diameter in each treatment group were recorded after four weeks. Shoot regeneration was recorded after eight weeks (all types of explants). During all experiments, glass jars were used with the 150 mL MS medium. Additionally, we used 4 explants per jar. All PGRs were purchased from Merck company.

Rooting and acclimatization: Regenerated explants (1–2 cm in length) were transferred to the MS (1/2 concentration) containing either 0, 0.1, or 0.2 mg/L IBA and NAA. Then, the MS contained NAA and IBA to induce rooting. After 8 weeks, the roots were counted in each plantlet. Well-rooted plantlets were transferred to an acclimatization medium, including cocopeat, perlite, sand, peat moss, and vermiculite, sterilized individually and mixed in equal proportions. Ultimately, they were transferred to the greenhouse (24 ± 3 °C and 70% relative humidity) to induce rooting in vivo. The plantlet survival percentage was calculated after 8 weeks. After obtaining complete acclimatization, the plantlets grew in sand and peat moss mixture (3:1).

Scanning electron microscopy (SEM) micrographs: The root explants were photographed by SEM electron microscopy during callus formation and shoot proliferation. After rapid freezing in liquid nitrogen, followed by storage (−80 °C), the samples were dried in the central laboratory of Shiraz University using a freeze dryer, and after metal coating, the explants were photographed as biological samples according to previous research [24]. In this research, SEM helps to observe the process of callus induction and regeneration more precisely.

### Statistical Analysis

Each experiment involved a completely randomized design (CRD). All data were analyzed using SAS 9.4 statistical software. Comparisons were outlined by mean values on the LSD test (*p* ≤ 0.05).

## 5. Conclusions

*Haworthia truncata*, along with other species in the genus, has gained significant popularity among cactus and succulent enthusiasts, particularly the bicolor cultivars. This species grew well without exogenously added PGRs and demonstrated potential for mass propagation through tissue culture. More so, *H. truncata* has been reported to form plantlets from all its vegetative and reproductive organs in vitro by the induction of callus from roots, leaves, and inflorescences. This led to desirable mutations in plants, especially in bicolor plants, which are highly demanded. Auxin requirements make this species different from other monocots. *H. truncata* may not follow the typical hormonal patterns observed in other monocots, making it an intriguing subject for further scientific exploration on its hormonal level at different developmental stages and in various explants. Further research regarding the micropropagation of other species of *Haworthia* is needed, including studies on color-controlling genes and genetic diversity.

## Figures and Tables

**Table 1:** Effects of indole 3, butyric acid (IBA), and 6, benzyl aminopurine (BA) on callus stimulation from inflorescence explants of *H. truncata*. The lower quantity in color and hardness has a higher quality.

Treatment	Concentration of PGRs (mg/L)		Diameter (cm)	Color	Hardness
	IBA	BA			
1	0	0	0.48 ^c^	2.65 ^b^	1.72 ^c^
2	0.5	-	1.24 ^b^	1 ^d^	1.07 ^e^
3	1	-	1.5 ^a^	1.62 ^c^	1.4 ^d^
4	1.5	-	1.67 ^a^	1.55 ^c^	1.27 ^de^
5	-	0.5	1.11 ^b^	2.4 ^b^	2.75 ^b^
6	-	1	1.16 ^b^	3.82 ^a^	3.75 ^a^

Similar letters denote insignificant differences per column (LSD test, *p* < 0.05). Color: 0 (White), 1 (Yellow), 2 (Yellow-green), 3 (Light green), 4 (Dark green). Hardness: 0 (Very soft, Water-soaked), 1 (Soft), 2 (Semi-hard), 3 (Hard), 4 (Very hard).

**Table 2:** Effects of 6, benzyl aminopurine (BA) indole 3, butyric acid (IBA) on shoot proliferation from callus inflorescence explants of *H. truncata* on BA-enriched media (1 mg/L).

Treatment	Concentration of PGRs (mg/L)		Number of Shoots	Shoot Length (cm)
	IBA	BA		
1	0	0	1.85 ^f^	0.19 ^c^
2	0.5	-	3.83 ^e^	0.65 ^ab^
3	1	-	9.97 ^a^	0.75 ^a^
4	1.5	-	5.22 ^bc^	0.54 ^b^
5	-	0.5	5.92 ^b^	0.21 ^c^
6	-	1	4.03 ^de^	0.2 ^c^
7	-	1.5	4.68 ^cd^	0.3 ^c^

Similar letters denote insignificant differences per column (LSD test, *p* < 0.05).

**Table 3:** Vitrification and deformation percentage in plantlets from callus.

Treatment	Concentration of PGRs (mg/L)		Vitrification (%)	Deformation (%)
	IBA	BA		
1	0	0	0 ^d^	0 ^c^
2	0.5	-	6.4 ^c^	0 ^c^
3	1	-	5.28 ^c^	0 ^c^
4	1.5	-	71.83 ^a^	0 ^c^
5	-	0.5	68.7 ^a^	0 ^c^
6	-	1	69.5 ^a^	14.54 ^a^
7	-	1.5	29.5 ^a^	4.93 ^b^

Similar letters denote insignificant differences per column (LSD test, *p* < 0.05).

**Table 4:** 6-Benzilaminopurine (BA) and auxin (IBA and NAA) affecting callus stimulation from leaf explants of *H. truncata*.

Treatment	Concentration of PGRs (mg/L)		Diameter (cm)	Color	Hardness
	BA	Auxin			
1	0	0	0.84 ^i^	1.89 ^j^	1.90 ^k^
2	0.25	0	1.55 ^c^	1.27 ^m^	1.26 ^n^
3		NAA 0.05	1.84 ^a^	1.66 ^k^	1.63 ^l^
4		NAA 0.25	0.33 ^q^	2.5 ^e^	2.46 ^gh^
5		IBA 0.05	0.73 ^m^	2.93 ^b^	3 ^c^
6		IBA 0.25	1.22 ^e^	2.07 ^i^	4 ^a^
7	0.5	0	0.82 ^j^	2.2 ^h^	2.42 h
8		NAA 0.05	0.87 ^h^	1.82 ^j^	2 ^j^
9		NAA 0.25	1.37 ^d^	2.39 ^f^	2.13 ^i^
10		IBA 0.05	1 ^g^	1.56 ^l^	1.54 ^m^
11		IBA 0.25	0.76 ^l^	2.33 ^fg^	3.35 ^b^
12	1	0	1.74 ^b^	2.48 ^e^	2.56 ^f^
13		NAA 0.05	0.22 ^r^	4 ^a^	3 ^c^
14		NAA 0.25	0.61 ^o^	2.83 ^c^	2.89 ^d^
15		IBA 0.05	0.65 ^n^	2.54 ^e^	4 ^a^
16		IBA 0.25	0.46 ^p^	3 ^b^	3.39 ^b^
17	1.5	0	1 ^g^	1 ^n^	1 ^o^
18		NAA 0.05	1.14 ^f^	2.7 ^d^	2.44 ^h^
19		NAA 0.25	0.78 ^k^	3 ^b^	2.5 ^g^
20		IBA 0.05	0 ^s^	0 ^o^	0 ^p^
21		IBA 0.25	0.65 ^n^	2.26 ^gh^	2.77 ^e^

Similar letters denote insignificant differences per column (LSD test, *p* < 0.05). Color: 0 (White), 1 (Yellow), 2 (Yellow-green), 3 (Light green), 4 (Dark green). Hardness: 0 (Very soft, Water-soaked), 1 (Soft), 2 (Semi-hard), 3 (Hard), 4 (Very hard).

**Table 5:** 6-Benzilaminopurine (BA) and auxin (IBA and NAA) affecting shoot proliferation from leaf explants of *H. truncata*.

Treatment	PGR Concentrations (mg/L)		Shoot Count	Shoot Length (cm)	Leaf Count
	BA	Auxin			
1	0	0	4.28 ^f–h^	0.89 ^ab^	3.86 ^a–e^
2	0.25	0	3.7 ^g–i^	0.69 ^c–f^	4.05 ^a–c^
3		NAA 0.05	2.66 ^ij^	0.79 ^bc^	4.72 ^a^
4		NAA 0.25	3.73 ^gl^	0.64 ^c–g^	3.5 ^b–g^
5		IBA 0.05	3.07 ^h–j^	1 ^a^	2.83 ^f–h^
6		IBA 0.25	0 ^k^	0 ^k^	0 ^k^
7	0.5	0	2.72 ^ij^	0.57 ^e–i^	3.83 ^a–e^
8		NAA 0.05	4.33 ^e–g^	0.33 ^j^	3.04 ^d–h^
9		NAA 0.25	5.66 ^de^	0.6 ^d–h^	2.92 ^e–h^
10		IBA 0.05	8.33 ^c^	0.52 ^f–i^	4.03 ^a–d^
11		IBA 0.25	2.5 ^ij^	0.42 ^h–j^	1.6 ^ij^
12	1	0	4.44 ^e–g^	0.72 ^b–e^	3.18 ^c–h^
13		NAA 0.05	10.33 ^b^	0.56 ^e–i^	3.17 ^c–h^
14		NAA 0.25	4.44 ^e–g^	0.66 ^c–g^	3.58 ^b–g^
15		IBA 0.05	6.33 ^d^	0.76 ^b–d^	4.44 ^ab^
16		IBA 0.25	3.33 ^g–i^	0.49 ^g–j^	3.25 ^c–g^
17	1.5	0	4.55 ^e–g^	0.41 ^ij^	2.62 ^gh^
18		NAA 0.05	0 ^k^	0 ^k^	0 ^k^
19		NAA 0.25	5.33 ^d–f^	0.44 ^h–j^	2.25 ^hi^
20		IBA 0.05	15 ^a^	0.46 ^h–j^	0.61 ^jk^
21		IBA 0.25	1.76 ^j^	0.69 ^c–f^	3.73 ^a–f^

Similar letters denote insignificant differences per column (LSD test, *p* < 0.05).

**Table 6:** 6-Benzilaminopurine (BA) and auxin (IBA and NAA) affecting callus stimulation from the root explants of *H. truncata*.

Treatment	Concentration of PGRs (mg/L)		Diameter (cm)	Color	Hardness
	BA	Auxin			
1	0	0	1.8 ^a^	3.08 ^bc^	2.75 ^cd^
2	0.25	0	1.14 ^cd^	2.1 ^ef^	1.67 ^i^
3		NAA 0.05	0.51 ^e^	2.43 ^de^	2.27 ^ef^
4		NAA 0.25	1.53 ^b^	1.65 ^g^	1.68 ^i^
5	0.5	0	0.65 ^e^	2.73 ^cd^	2.96 ^c^
6		NAA 0.05	1.65 ^ab^	1.84 ^fg^	1.78 ^hi^
7		NAA 0.25	0.96 ^d^	2.54 ^de^	2.5 ^de^
8		IBA 0.05	0.54 ^e^	3 ^bc^	4 ^a^
9	1	0	1.14 ^cd^	2.14 ^ef^	1.90 ^g–i^
10		NAA 0.05	1.16 ^cd^	2.1 ^ef^	2.14 ^fg^
11		NAA 0.25	0.62 ^e^	3.09 ^bc^	2.57 ^d^
12	1.5	0	1.25 ^c^	3.36 ^b^	3.4 ^b^
13		NAA 0.05	1.11 ^cd^	2.54 ^d^	2.29 ^ef^
14		NAA 0.25	0.69 ^e^	2.96 ^c^	1.96 ^gh^
15		IBA 0.05	0.96 ^d^	3.9 ^a^	3.76 ^a^

Similar letters denote insignificant differences per column (LSD test, *p* < 0.05). Color: 0 (White), 1 (Yellow), 2 (Yellow-green), 3 (Light green), 4 (Dark green). Hardness: 0 (Very soft, Water-soaked), 1 (Soft), 2 (Semi-hard), 3 (Hard), 4 (Very hard).

**Table 7:** 6-Benzilaminopurine (BA) and auxin (IBA and NAA) affecting shoot proliferation from root explants of *H. truncata*.

Treatment	Concentration of PGRs (mg/L)		Shoot Count	Shoot Length (cm)	Leaf Count
	BA	Auxin			
1	0	0	1 ^g^	1 ^a^	4.16 ^a^
2	0.25	0	1 ^g^	0.25 ^e^	1.16 ^f^
3		NAA 0.05	2.6 ^f^	0.29 ^de^	3.03 ^bc^
4		NAA 0.25	3.06 ^e^	0.33 ^cd^	1.65 ^e^
5		IBA 0.25	3 ^e^	0.37 ^c^	2.62 ^b–d^
6	0.5	NAA 0.25	5.7 ^b^	0.35 ^cd^	2.78 ^b–d^
7		IBA 0.05	3.96 ^d^	0.54 ^b^	3 ^bc^
8		IBA 0.25	3.16 ^e^	0.35 ^cd^	3.06 ^b^
9	1	0	5 ^c^	0.28 ^de^	2.6 ^cd^
10		NAA 0.05	7.5 ^a^	0.24 ^e^	2.33 ^d^
11	1.5	0	4.03 ^d^	0.25 ^e^	2.5 ^d^

Similar letters denote insignificant differences per column (LSD test, *p* < 0.05).

**Table 8:** Concentrations of 0.1 and 0.2 mg/L IBA and NAA compared to the control treatment, affecting the number, size, and diameter of roots, and percentage of root formation.

Treatment	Concentration of PGRs (mg/L)		Root Length (cm)	Diameter of Root (mm)	Number of Roots	Root Percentage
1		0	3.04 ^a^	0.14 ^a^	1.33 ^b^	37.5 ^b^
2	NAA	0.1	1.95 ^ab^	0.17 ^a^	1.83 ^b^	50 ^ab^
3		0.2	3.20 ^a^	0.25 ^a^	2.08 ^b^	75 ^a^
4	IBA	0.1	2.81 ^a^	0.31 ^a^	7.16 ^a^	62.5 ^ab^
5		0.2	0.63 ^b^	0.18 ^a^	5.77 ^a^	56.25 ^ab^

Similar letters denote insignificant differences per column (LSD test, *p* < 0.05).

**Table 9:** Concentrations of 1 and 1.5 mg/L of plant growth regulators NAA and IBA compared to the control (MS medium without PGRs) treatment, affecting the number, size, and diameter of roots, and percentage of root formation.

Treatment	PGRs (mg/L)		Root Length (cm)	Root Diameter (mm)	Root Number	Root Percentage
1		0	3.04 ^a^	0.14 ^a^	1.33 ^b^	37.5 ^c^
2	NAA	1	0.70 ^b^	0.24 ^a^	4.41 ^ab^	37.5 ^c^
3		1.5	0.70 ^b^	0.24 ^a^	6.33 ^ab^	43.75 ^c^
4	IBA	1	1.59 ^b^	0.28 ^a^	8.83 ^ab^	75 ^b^
5		1.5	1.34 ^b^	0.17 ^a^	6.44 ^ab^	100 ^a^

Similar letters denote insignificant differences per column (LSD test, *p* < 0.05).

**Table 10:** Acclimation affecting root development among the plantlets, 8 weeks after transfer to pots.

Treatment	Acclimation Mediums	Root Length (cm)	Root Number	Leaf Number	Plant Length	Rootlet (%)	Root Rot (%)
1	sand	1.77 ^b^	1.25 ^c^	1 ^bc^	0.85 ^a^	25 ^ab^	0 ^b^
2	cocopeat	0.80 ^cd^	2.25 ^b^	1.25 ^bc^	0.92 ^a^	21.31 ^ab^	26.49 ^a^
3	Perlite	0.90 ^c^	3.75 ^a^	3 ^a^	1.27 ^a^	35.42 ^ab^	13.54 ^ab^
4	Perlite + cocopeat	2.40 ^a^	0.72 ^d^	1.25 ^bc^	1 ^a^	40.47 ^a^	11.3 ^ab^
5	Sand + Perlite	1.48 ^b^	1.25 ^c^	0.25 ^c^	1 ^a^	4.17 ^b^	13.33 ^ab^
6	Peatm + Perlite	0.61 ^c–e^	0.05 ^e^	1.75 ^ab^	0.75 ^a^	36.31 ^a^	25.59 ^a^
7	Peatm + Perlite + vermi	0.31 ^de^	0.45 ^de^	0.25 ^c^	1 ^a^	15.83 ^ab^	15.83 ^ab^
8	Sand + Perlite + cocopeat	0.27 ^de^	0.15 ^e^	0.25 ^c^	0.5 ^a^	15 ^ab^	25.91 ^a^
9	Sand + Peat moss + Perlite	0.20 ^e^	0.15 ^e^	0 ^c^	1.5 ^a^	22.92 ^ab^	11.31 ^ab^

Similar letters denote insignificant differences per column (LSD test, *p* < 0.05).

## Data Availability

Data is available via sending an email to the corresponding author. The data are not publicly available due to Shiraz University policy.
